# Biodiversity of Phototrophs and Culturable Fungi in Gobustan Caves

**DOI:** 10.3390/life13010164

**Published:** 2023-01-05

**Authors:** Svetlana Evgenievna Mazina, Tatiana Vladimirovna Gasanova, Ekaterina Vitalievna Kozlova, Anna Vladimirovna Popkova, Anton Sergeevich Fedorov, Irina Leonidovna Bukharina, Anna Sergeevna Pashkova, Maxim Viktorovich Larionov, Rahman Rahim oglu Abdullayev, Vugar Urfat oglu Isaev

**Affiliations:** 1 Research and Technical Centre of Radiation-Chemical Safety and Hygiene FMBA of Russian Federation, 40 Schukinskaya Street, 123182 Moscow, Russia; 2 Peoples Friendship University of Russia (RUDN University), 6 Miklukho-Maklaya Street, 117198 Moscow, Russia; 3 Federal State Budgetary Educational Institution of Higher Education “State University of Land Use Planning” (SULUP), 15 Kazakov Street, 105064 Moscow, Russia; 4 Lomonosov Moscow State University, 1-12 Leninskiye Gory, GSP-1, MSU, 119991 Moscow, Russia; 5 Udmurt State University, 6 Universitetskaya Street, 426034 Izhevsk, Russia; 6 Russian State Social University (RSSU), 4 Wilhelm Peak Street, Building 1, 129226 Moscow, Russia; 7 State University of Management (SUM), 99 Ryazanskij Prospect Street, 109542 Moscow, Russia; 8 World-Class Scientific Center “Agrotechnologies for the Future” (CAAT), Russian State Agrarian University-Moscow Timiryazev Agricultural Academy, 49 Timiryazevskaya Street, 127550 Moscow, Russia; 9 Gobustan National Historical-Artistic Preserve, Gobustan Settlement, AZ1080 Baku, Azerbaijan

**Keywords:** biodiversity, composition and state of biocenoses, subterranean habitats, biofilms, cyanobacteria, algae, mosses, microfungi, caves, grottoes, environmental factors, Gobustan National Historical and Artistic Preserve

## Abstract

Unique natural objects, such as the caves of the Gobustan National Historical and Artistic Preserve, are also of great cultural and historical value due to rock art and sites of ancient people. A favorable microclimate makes these habitats convenient for colonization by microbiota, including phototrophs. In arid regions with intense seasonal fluctuations of microclimatic parameters, the conditions for survival are the least favorable; therefore, it becomes especially important to determine the composition of communities that are the most adapted to specific conditions. This work aimed to identify the biodiversity of communities of caves and grottoes of the Gobustan Reserve. The studies were carried out in July 2019. Samples were analyzed for cyanobacteria and algae by microscopy and cultivation methods, microfungi were isolated by soil dilution, and the fouling glass method was also used. In total, 29 taxa of cyanobacteria and algae, 18 taxa of fungi, and 3 species of mosses were identified. The studied habitats were dominated by the algae *Chlorella vulgaris*, *Aphanocapsa* sp., and *Stichococcus bacillaris*; the subdominants were *Jaaginema subtilissimum*, *Leptolyngbya tenuis*, *Chlorococcum minutum*, and *Humidophila contenta*. Microfungi had the highest occurrence of *Aspergillus niger*, *Aureobasidium pullulans*, *Alternaria alternata*, and *Talaromyces ruber*. It was noted that cyanobacteria dominated in morphologically differentiated biofilms and green algae on the rocks. The greatest number of microfungi was found in the aphotic zone and bryophyte tufts. The dominance of green algae is atypical for most caves of other regions and may be associated with intense lighting of habitats. The absence of protonema is a consequence of the aridity and low moisture content of the substrates.

## 1. Introduction

The Gobustan National Historical and Artistic Preserve includes a large complex of unique historical sites. It is located in Azerbaijan, in the territory of the Karadag and Apsheron regions. This area is a plain between the southeastern slope of the Great Caucasus Range and the Caspian Sea. In 2007, this area was declared a UNESCO World Heritage Site. The sites of ancient people with rock art are of great historical value [1]. Many petroglyphs are in caves and grottoes, where the special microclimate is formed. Unlike the surface, it is characterized by increased humidity and limited influence of precipitation and air currents. In such conditions, the preservation of drawings can be better than in the open areas. However, these habitats are actively colonized by microbiota, bryophytes, and ferns.

Such lithobiont communities are typical for the entrance zone of many caves and grottoes. Phototrophs in illuminated (photic) areas are usually distributed in mosaic areas and consist of the separate communities with distinct dominants. Autotrophic and heterotrophic species are combined in the mentioned communities, which allows us to consider them as a consortium [2]. The presence of spatial and trophic links provides community resilience not only to changes in the microclimate but also to the lack of light [3].

In arid regions, a significant factor determining the distribution, biodiversity, and physiological activity of organisms in the photic zones of caves and grottoes is a seasonal variation in humidity and temperature. Under stressful conditions, the emergence of environmentally resistant species and the adaptation at the community level can be expected. This is confirmed by several studies of caves and grottoes, revealing a regional trend of cave biodiversity similarity. The mentioned study was based on a comparison of the species composition of the photic zone [4], and the influence of entrance morphology on species composition [5]. It is interesting to note that a comparison of the species composition of cave algoflora from different regions reveals a group of species belonging to cosmopolites and ubiquitous species characteristic of this habitat [6]. The analysis of the species composition and community structure of caves and grottoes in arid territories will make it possible to determine the role of such species in communities and their adaptations limits.

Cave entrance areas are considered to be ecotones [7]. The named areas are relatively isolated from surface influences, photic zone communities can be considered as a relict. Their development is at the stage of a climax phase of succession [8]. The territory of Gobustan undergoes a minimal anthropogenic impact, which responds to the conditions of natural reserve. Therefore, the communities of photic zones are in their native state and reflect adaptations to the arid conditions. The last point is that no studies of the cave biota have been carried out on the territory of Gobustan, which further compels the relevance of the research.

The work aims to identify the biodiversity of the lithobiont communities of the caves of Gobustan.

## 2. Materials and Methods

### 2.1. Study Area

The Gobustan National Historical and Artistic Preserve is located in the subtropical belt with a climate of semideserts and dry steppes, moderate winters, and dry hot summers. The average annual temperature is 12–14.5 °C.

**Figure 1:**
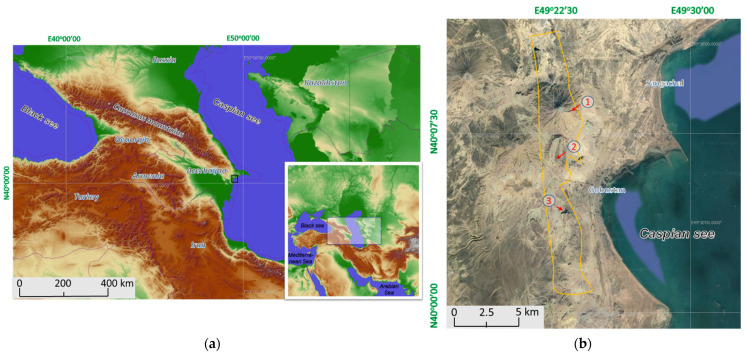
(**a**) Location of Gobustan National Historical and Artistic Preserve; (**b**) Gobustan samplings areas: 1—Kanizadag mountain, 2—Beyuk-Dash Mountain, 3—Kichik-Dash Mountain.

The caves and grottoes of the Gobustan reserve were surveyed in July 2019, the sampling areas are shown in Figure 1, and the description of the samples is given in Table 1.

### 2.2. Methods of Phototrophic Analysis

Samples of bryophytes and algae from the walls and the floor of caves were collected in sterile vials. A total of 10 samples were collected. Algae and cyanobacteria were cultured using Gromov’s medium No. 6 and Bristol’s liquid culture mediums. Sections of lithobiont communities, substrates, and moss tufts were placed in liquid culture medium to obtain an algal accumulation culture. In addition, we used gel agarose media on which prints were made by applying pieces of rock, soil with phototrophs, or tufts of mosses. Bristol medium was modified by Hollerbach for soil algae composition (g/L): NaNO_3_—0.25; KH_2_PO—0.25; MgSO_4_·7H_2_O—0.15; CaCl_2_—0.05; NaCl—0.05; Fe_2_Cl_6_—traces (3 drops of 1% solution). Medium No. 6. composition (g/L) KNO_3_—1; K_2_HPO_4_—0.2; MgSO_4_·7H_2_O—0.2; CaCl_2_—0.15; NaHCO_3_—0.2; micronutrient solution 1ml/l. Solution of trace elements (g/L): ZnSO_4_·7H_2_O—0.022; MnSO_4_—1.81; CuSO_4_·5H_2_O—0.079; NaBO_3_·4H_2_O—2.63; (NH_4_)_6_Mo_7_O_24_·4H_2_O—1.0; FeSO_4_·7H_2_O—9.3; CaCl_2_—1.2; Co(NO_3_)_2_·H_2_O—0.02; Trilon B (EDTA)—10.0.

Illumination intensity was 2 × 10^19^ m^−2^s^−1^. The exposure temperatures of the stored cultures were 12, 24, and 37 °C for the best development of all species. Pure cultures were isolated on gel media at room temperature.

Phototrophic communities were examined using a Leica DMLS light microscope (Wetzlar, Germany, Leica Microsystems). Samples were prepared by separating small fragments from communities (biofilms) and placing them in water droplets. The abundance of species during cultivation was also assessed on a 5-point scale. The following scale was used to determine the abundance of microscopic species during microscope viewing (every sample was investigated using discreet sample areas, microscope fields of view): 1—species occurred singularly in microscope field of view or in single areas of an overgrowth spot; 2—species occurred singularly in 20 to 50% of the microscope fields of view; 3—species occurred in small numbers in 50% of the microscope fields of view; 4—species occurred in large numbers in 50% of the microscope fields of view; 5—species occurred in large numbers in each microscope field of view. Diatom algae were identified using scanning electron microscopy (CamScan). Culturing mixed cultures often causes problems with diatom algae isolation. Therefore, when algae were detected in a sample, it was prepared for scanning microscopy. Parts of the sample on a slide were successively washed with hydrogen peroxide and water, dried, and prepared for SEM viewing.

The species were identified using several keys [9,10,11,12,13,14]. Systematics of cyanobacteria and algae is provided by https://www.algaebase.org (accessed on 27 December 2022). database [15] and the mosses by Ignatov and Ignatova [16].

### 2.3. Analysis of Microfungi

Microfungi were isolated from clay sediments samples by the soil dilution method of Vaksman in the modification of Zvyagintsev with seeding and counting on solid agar nutrient media and release into pure cultures [17]. Soil samples were treated using the standard method, suspended, and aliquots were seeded from serial dilutions of the suspension on the surface of nutrient agar. Additionally, the fouling glasses method was applied. Specifically, the studied substrates were placed in a Petri dish and covered with coverslips, pressing them slightly to the substrate. Then, Petri dishes were placed in an environmental chamber; the glasses were viewed at intervals of 7 days. In addition, prints of the communities were made on nutrient media. Tufts of mosses and algal and cyanobacterial communities were applied to the medium and lightly pressed to obtain a print for microfungal analysis on the surface of the communities [18].

Rock samples were placed in a sterile isotonic NaCl solution and treated with ultrasound, after which an aliquot of the suspension was seeded onto a selective medium. For the cultivation of microfungi, Czapek-Dox agar (sucrose concentration of 15 and 0.3%) and potato glucose agar were used [18].

Cultivation of microfungi was carried out at a temperature of 12, 24, and 37 °C, and calculation of the grown colonies and isolation of pure cultures was carried out every week. Identification of microfungi was performed according to [19,20,21,22,23,24]. Species names are given according to the http://www.mycobank.org (accessed on 1 December 2022) database [25].

The frequency of occurrence of individual species was determined as the ratio of the sum of samples in which the species were detected to all samples.

### 2.4. Analysis of Substrate

Substrate samples (clay sediments and limestone) were analyzed for several physical and chemical parameters. The lowest moisture capacity of substrates, pH of aqueous suspension, and the carbonates content were determined by titrimetric method [26].

### 2.5. Statistical Analysis

To determine the dominant algal taxa in each sample, an index of relative abundance expressed in percentage was used. The similarity of algal and fungal species in different caves and substrates was compared using the Jaccard index. A value greater than 0.4 was considered a reference similarity [27]. The similarity of the biodiversity between the studied sites was assessed using the Euclidean distance.

## 3. Results

**Figure 2:**
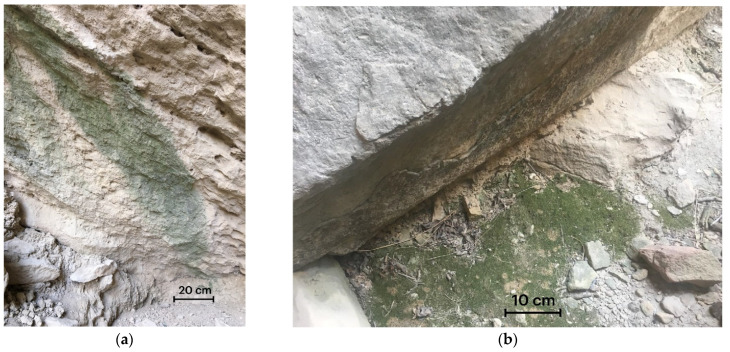
(**a**) Biofilms on the grotto walls; (**b**) Bryophytes on the grotto walls.

Green, blue-green, or greenish-brown lithobiont communities, including algae, cyanobacteria, and bryophytes, were observed on cave walls (Figure 2a). Phototrophs (algae or cyanobacteria) were identified in all examined samples during their cultivation, including in the form of rudiments, which were collected in the aphotic zone with no visible communities.

A total of 29 taxa of algae and cyanobacteria were identified, of which 12 belong to *Cyanobacteria*, 12 belong to *Chlorophyta*, 4 belong to *Bacillariophyta*, and 1 belongs to *Charophyta*; the list of taxa and their distribution are shown in Table 2, and the illustrations are shown in Figures S1 and S2.

The studied habitats were dominated by the species Chlorella vulgaris, *Aphanocapsa* sp., Stichococcus bacillaris, and the subdominants were Jaaginema subtilissimum, Leptolyngbya tenuis, Chlorococcum minutum, and Humidophila contenta.

Among the microscopic fungi isolated from grottoes and caves, the largest number of species belonged to the genera *Aspergillus* and *Penicillium*. The most frequently identified species in the samples were *A. niger*, *Aureobasidium pullulans*, *Alternaria alternata*, and *Talaromyces ruber* (Table 3). The species *Chaetomium globosum* and *P. cyclopium* were absent in samples from the photic zone and *Fusarium* sp. was absent in samples from the aphotic zone. The total number of species in the sampling points was small, with 2–10 in the photic zones and 7–10 in the aphotic zones.

Three species of bryophytes were identified, *Didymodon* sp., *Tortula* sp., and *Sciuro-hypnum* sp., which formed tufts and played an environment-forming role in lithobiont communities (Figure 2b). In each habitat, the moss tufts were monospecific. *Didymodon* sp. was found at points 1 and 2, *Tortula* sp. at points 5 and 6, and *Sciuro-hypnum* sp. at point 4.

Our experience with culture isolation has shown that some species are often lost when cultured at room temperature. The cave temperature is usually almost equal to the local yearly average temperature [28]. In this study, based on the location of the caves, we hypothesized that there may be fluctuations in temperatures throughout the year in the entrance zone. Therefore, the cultivation of algoflora and isolation of microfungi were carried out at three temperatures. The abundance of algal taxa in culture and in the native community was similar at cultivation temperatures of 24 and 27 °C but different at 12 °C (Table S1). Particularly interesting was the increase in the abundance of all species in samples from the photic zones of the caves and the abundance of most of the dominant species.

Only some species of microfungi were identified at three temperatures: *A. ochraceus*, *Aureobasidium pullulans*, *Emericella usta*, and *P. chrysogenum*. The ability to grow at 37 °C may indicate the possibility of development in the human body and potential pathogenicity.

The characteristics of the studied substrates had no significant differences between the photic and aphotic zones (Table 4). The aqueous extracts from clay sediments were slightly alkaline, as were the aqueous extracts from limestones. Clay sediments had a high moisture-holding capacity.

The similarity in the biodiversity of different habitats was evaluated separately for algae and microfungi (Table 5). Algae had reference similarities: in mosses tufts (points 4, 6, and 7), with points 6 and 7 from the same cave; in biofilm communities (points 1 and 8 and points 2 and 8), with points 1 and 2 being close to the reference similarity. Microfungi were similar only in communities dominated by mosses (points 6 and 4); point 4 was similar to aphotic zones 9 and 10 and points 6 to 10. Interestingly, biofilms dominated by cyanobacteria and algae were similar in microfungal composition (points 1 and 8), while biofilms of green algae were completely different (points 1 and 2). Microfungi from the moss communities of point 7 were similar to the fungi of the aphotic zones of points 3 and 5. The microfungi of the aphotic zones of points 10 and 9 were also similar. Analysis of the communities using the Euclidean metric showed the association of the aphotic zones of part of the caves’ illustrations in Figure S3.

## 4. Discussion

Many caves are dominated by cyanobacteria [4,6,29,30], but in Gobustan the number of species of green algae and cyanobacteria was the same. A similar distribution was found in a number of Polish caves [31,32].

Grottoes and small cavities are usually better illuminated than caves, due to the peculiarities of the morphology of the entrances. The dominance of green algae in most studied habitats may be associated with a high level of illumination. This corresponds to the study that was carried out in the grottoes of the national nature park “Podilsky Tovtry” (Ivano-Frankivsk Oblast, Ukraine) [33]. Cyanobacteria dominated in the aphotic zones. In our study, the light level was not assessed and should be clarified in the future.

The dominance of *Chlorella* and filamentous cyanobacteria in epilithic phototrophic communities is characteristic of many Cango caves in southern Africa [34], Katerinska cave in the Czech Republic [35], and karst caves in Slovenia [36].

The photic zones of caves are characterized by continuity of phytocenoses but consist of discrete communities whose formation is related to landscape features and illumination [8,37]. At the same time, it was noted that at low illumination levels, cyanobacteria dominate, and cyanobacteria predominate in the species composition of photic zones and lampenflora [38]. However, in some cases, the dominance of green algae was revealed in the entrance zones [4,33], and it has been suggested that green algae predominate under conditions of low humidity and high illumination, which depends on the morphology of the cave entrance [5].

Studying the grottoes of “Podilsky Tovtry”, it was noted that the species diversity was higher in shaded areas, which can be attributed to less evaporation and higher rock moisture in conditions of low insolation [33]. This trend should be typical for grottoes and small caves, especially for arid regions, where, due to high temperature and low humidity, caves and grottoes function as a refugium for species that can survive in low light and need high humidity, such as algae, cyanobacteria, some bryophytes, and ferns. Observations carried out in Montenegro have demonstrated the absence of ferns in the photic zones of caves [5,37], while in the caves of the Caucasus region, ferns are an obligatory component of the vegetation of the caves entrance zone [8,39,40]. This can be associated with higher temperatures, less precipitation in the summer season, and lower humidity in Montenegro, where precipitation rates are decreasing [41]. No ferns were found in the entrance zones of the caves and grottoes of Gobustan, which confirms the decrease in the number of species that need high humidity in the photic zones of caves in an arid climate. In the caves of Gobustan, moss protonema was absent in the phototrophic communities, although it is usually found in most communities and may occupy a dominant position [5,8,39]. We can assume that we were unable to identify the protonema due to sampling during the dry period of the year.

Considering each examined sample of Gobustan caves, cyanobacteria dominated in the aphotic zones, which agrees with the statement about the predominance of cyanobacteria in low light [6,33,34]. The total number of phototrophic species in these samples in three caves was six, and only one sample had nine species. In photic zones, the number of species varied from 11 to 18. That is, the number of species in the illuminated areas was slightly higher. An unnamed cave near Kanizadag mountain was an exception, with seven species found and dominated by cyanobacteria *Leptolyngbya tenuis*, which forms biofilms on the substrate surface, which may prevent the development of other algae. It was noted that in samples from aphotic zones the species abundance was more uniform than in samples from photic zones: one or two species dominated, and the representation in the community of other species was the same. Another cave, Beyuk-Dash (“Bol’shoj Kamen”) Cave, dominated by cyanobacteria, was also characterized by a predominance of biofilm-forming species, *Leptolyngbya foveolarum*, *Leptolyngbya tenuis*, and *Aphanocapsa* sp., but total species abundance was higher in the photic zone (Table 3).

For Gobustan caves located in the arid region, the dominance of green algae and cyanobacteria was revealed in different communities, which confirms the ambiguity of conclusions about the dominance of any one group of algae in the communities of photic zones and presents questions about the prevailing factor influencing the formation of the biodiversity of these communities.

Many genera and species of cyanobacteria that are characteristic of caves have been found in Gobustan caves: *Nostoc*, *Lyngbya* [42], *Jaaginema*, *Leptolyngbya foveolarum*, *Microcoleus* [42,43,44], *Nostoc*, *Leptolyngbya* [45]. Usually, in the flora of caves there is a high diversity of unicellular colonial cyanobacteria, representatives of Chroococcales Chroococcidiopsidales, genera *Gloeocapsa*, *Gloeothece*, *Aphanocapsa*, *Chroococcidiopsis*, *Gloeocapsa*, *Gloeocapsopsis*, *Synechococcus* are found [44,46]. However, in Gobustan filamentous, cyanobacteria predominate.

In the studied caves, only one biofilm was blue-green in color and its structure was dominated by cyanobacteria. Cyanobacteria dominated in moss tufts, where light levels were lower than on open surfaces. This observation is consistent with the data that show the amount of green algae decreases in caves and the diversity of cyanobacteria increases in darker and deeper areas [47].

For the entrance areas of the caves, if we accept the hypothesis of their long-term stable development under conditions of insignificant variations in the ecotope, we can assume a stable state of communities and the final stages of succession. In this case, changes in the species composition can only be associated with stress factors. Cave studies conducted in southwestern China confirmed the microrefugia role of cave entrance zones, and the flora of the photic zones was determined to be a relic of the regional karst forest flora [48]. The identified algoflora in the caves of Gobustan can be interpreted as lithophilic communities, characteristic of caves, as species enduring seasonal adversity in the caves, and even relict species.

Microfungi were found in all studied habitats. The highest number of species was in the genera *Aspergillus* and *Penicillium*, as was observed in most caves [49,50,51]. Frequently occurring species are common in caves. *Aureobasidium pullulans* is an endophytic fungus common in various caves [50,52], including ice and volcanic caves, and is a primary colonizer of oligotrophic environments [53,54]. *A. niger* may exhibit keratinolytic activity [55], and *Alternaria alternata* may have lignolytic potency [56]. Microfungi *A. niger*, *Alternaria alternata*, and *P. chrisogenum* are found in native cave species not visited by humans [57] and are listed as a part of the microbiota of many other caves [50,58,59,60,61]. The low diversity of fungi in caves is associated with an oligotrophic environment [50,54] and the caves of Gobustan are not an exception.

From the soil samples, the greatest diversity of fungi is obtained at a temperature of 10 °C; compared with higher temperatures, increased humidity and habitat temperature lead to an increase in the proportion of potentially pathogenic microfungal species [62]. It can be assumed that in caves in southern regions located at low altitudes, the conditions for the development of pathogenic species are the most favorable. Species that we isolated at 37 °C are pathogenic [63].

Our study was conducted in caves, analyzing the species composition of fungi of different cave zones and comparing them with the microbiota of soil on the surface. The separation of species that are in different functional states and assessment of their importance in communities is a challenging point. To date, there is evidence that the biodiversity of fungi in the deep zone of the cave and on the surface is similar [60]. The entrance zone is characterized by a relationship between the species composition of fungi and habitat diversity [64]. In this study, we could not reliably confirm the similarity of the microfungal communities of the photic and aphotic zones, although this seems logical for small cavities and grottoes whose morphology provides an unobstructed exchange with the surface. However, we found a similarity between the communities of aphotic zones of some caves and the communities of mosses and biofilms.

It can be assumed that a comparative analysis of the mycoflora of cave entrances in arid regions and a study of the adaptation of microfungi to the cave conditions will make it possible to identify species that are indicators of climate aridization. Compared to the entrance areas of caves and grottoes of other regions, the biodiversity in Gobustan is lower.

The species composition and structure of phototrophic communities are described for the first time for Gobustan caves. These data are useful for the conservation of the biodiversity of the reserve. Since there are no unambiguous opinions on the prevailing factors of species composition formation and dominant species in communities of photic zones of caves, it is planned to continue such research on the example of Gobustan. Regional differences in the composition of cave communities suggest the need to develop an algorithm for assessing the spectrum of significant factors in the formation of the biodiversity of photic zones, which is especially important for arid regions.

## 5. Conclusions

A study of phototrophs and cultured microfungi in six small caves of the Gobustan National Historical and Artistic Preserve was carried out for the first time. Among phototrophs, only three species of mosses were identified and covered-seeded; ferns and their sprouts were absent in the caves. Cyanobacteria dominated in the aphotic zones of caves and in moss tufts, while green algae mainly dominated in biofilms in photic zones.

Unlike many studies on the biodiversity of phototrophic communities in caves, green algae dominate in Gobustan and there is practically no moss protonema in the substrates. This situation may reflect the aridity of the climate and the low humidity of air and substrates in the habitats of communities. The largest amount of microfungi was found in the aphotic zone and bryophyte tufts. Low species richness may be an indication of aridity.

Characteristic species that dominated in the studied caves: *Chlorella vulgaris*, *Aphanocapsa sp*., and *Stichococcus bacillaris*; the subdominants were *Jaaginema subtilissimum*, *Leptolyngbya tenuis*, *Chlorococcum minutum*, and *Humidophila contenta*.

Microfungi are represented by species that were also found in caves in other studies. The biodiversity of microfungi was low.

The comparison of biodiversity in different communities and habitats using the Jaccard index revealed a number of trends: the species-specificity of algae and microfungi in moss communities, the similarity of biofilm communities regardless of dominant species, and the similarity of microfungi species in aphotic zones and in moss communities. Despite the identified similarities, the co-communities of cave entrance areas of the photic and aphotic zones of each cave have a unique species composition. The information obtained is useful in strengthening nature protection work in relation to the described and other similar natural and territorial complexes.

## Figures and Tables

**Table 1:** Sampling points characteristics.

No.	Location of Sampling Point	Sample Type
1	Beyuk-Dash («Bol’shoj Kamen’») Cave, located around Beyuk-Dash Mountain.	Blue-green thin biofilms on the surface of clay deposits.
2	Kichik-Dash («Malen’kij Kamen’») Cave, located around Kichik-Dash Mountain.	Dark green thin biofilms on the surface of limestone rock.
3	An unnamed cave, located near Beyuk-Dash Mountain. The site of an ancient hearth.	Clay sediments without phototrophs (aphotic zone).
4	An unnamed cave, located near Beyuk-Dash Mountain.	Greenish-brown or green bryophytes on the surface of clay deposits.
5	An unnamed cave, located near Beyuk-Dash Mountain. The parking lot of an ancient man.	Clay sediments without phototrophs (aphotic zone).
6	An unnamed cave (grotto), near Kanizadag mountain.	Green mosses tufts on the surface of limestone rock.
7	An unnamed cave, near Kanizadag mountain. An area near to the ancient rock paintings.	Greenish-brown or green bryophytes on the surface of limestone rock.
8	An unnamed cave, near Kanizadag mountain. An area near to the ancient rock paintings.	Dark green thin biofilm growth on the surface of limestone rock.
9	New small cave located near Kichik-Dash Mountain.	Clay sediments without phototrophs (aphotic zone).
10	A cave around Yazili Tepe rocks near Kanizadag mountain.	Clay sediments without phototrophs (aphotic zone).

**Table 2:** Relative abundance of taxa.

Taxon	Relative Abundance, %										
Sample Point	1	2	3	4	5	6	7	8	9	10	Total
Phylum Cyanobacteria											
*Aphanocapsa* sp. Nägeli	10.8	8.3	25	10	-	9.5	-	5.9	-	20	8.4
*Cyanothece aeruginosa* (Nägeli) Komárek	8.1	8.3	-	-	-	-	-	2.9	-	-	3.2
*Jaaginema subtilissimum* (Kützing ex Forti) Anagnostidis and Komárek	2.7	8.3		5	25			5.9		10	4.7
*Leptolyngbya* sp. Anagnostidis and Komárek	5.4	-	25	-	-	-	-	5.9	-	-	3.2
*Leptolyngbya foveolarum* (Gomont) Anagnostidis and Komárek	10.8	8.3	-	-	-	-	-	5.9	-	-	4.2
*Leptolyngbya tenuis* (Gomont) Anagnostidis and Komárek	10.8	-	12.5	-	-	-	-	2.9	28.6	10	4.8
*Lyngbya nigra* C. Agardh ex Gomont	5.4	-	12.5	-	-	-	-	2.9	-	-	2,1
*Microcoleus autumnalis* (Gomont) Strunecky	5.4	-	-	5	-	-	-	-	-	10	2.1
*Nostoc microscopicum* Carmichael ex Bornet and Flahault	-	-	-	5	-	9.5	0.05	-	14.3	-	2.6
*Nostoc punctiforme* Hariot	-	-	-	10	-	14.3	0.15	-	-	-	4.2
*Pseudanabaena limnetica* (Lemmermann) Komárek	2.7	-	-	-	12.5	-	-	-	-	10	1.6
*Schizothrix* sp. Kutzing ex Gomont	-	4.2	-	-	-	-	-	-		-	0.5
Phylum Bacillariophyta											
*Craticula cuspidata* (Kutzing) D.G.Mann	2.7	-	-	5	-	-	-	-	-	-	1.1
*Hantzschia amphioxys* (Ehrenberg) Grunow	2.7	-	-	5	-	4.8	-	-	-	-	1.6
*Humidophila contenta* (Grunow) R.L.Lowe et al.	5.4	-	12.5	10	-	4.8	10	2.9	-	-	4.7
*Luticola mutica* (Kützing) D.G.Mann	2.7	-	-	-	-	-	0.5	-	-	-	1.1
Phylum Chlorophyta											
*Apatococcus lobatus* (Chodat) J.B.Petersen	-	-	-	-	-	4.8	-	5.9	14.3	-	2.1
*Chlorella vulgaris* Beijerinck	8.1	20.8	12.5	15	25	19	15	14.7	14.3	-	14.2
*Chlorococcum infusionum* (Schrank) Meneghini	2.7	12.5	-	-	-	4.8	-	2.9	-	10	3.7
*Chlorococcum minutum* R.C.Starr	8.1	4.2	-	5	-	4.8	5	5.9	-	-	4.7
*Chloroidium ellipsoideum* (Gerneck) Darienko et al.	-	-	-	-	-	-	5	5.9	-	-	1.6
*Chlorokybus atmophyticus* Geitler	2.7	-	-	-	-	4.8	-	-	-	-	1.1
*Desmococcus olivaceus* (Persoon ex Acharius) J.R.Laundon	-	8.3	-	-	-	9.5	5	2.9	-	-	3.2
*Elliptochloris subsphaerica* (Reisigl) Ettl and G.Gärtner	-	8.3	-	-	12.5	-	-	8.8	14.3	10	4.2
*Gongrosira terricola* Bristol	-	-	-	-	-	-	10	-	-	10	1.6
*Neochlorosarcina minor* (Gerneck) V.M.Andreyeva	-	-	-	-	12.5	-	10	5.9	-	-	2.6
*Neochlorosarcina* sp. S.Watanabe	-	-	-	5	-	-	-	5.9	-	-	1.6
*Stichococcus bacillaris* Nägeli	2.7	8.3	-	10	12.5	9.5	10	5.9	14.3	10	7.4
Phylum Charophyta											
*Klebsormidium* sp. PCSilva	-	-	-	10	-	-	5	-	-	-	1.6

**Table 3:** The occurrence of microfungi at sampling points and the temperatures at which the microfungi were isolated.

Taxon/Sample Point	1	2	3	4	5	6	7	8	9	10
*Alternaria alternata* (Fr.) Keissl.	24.37		24.37		24.37			24.37	24.37	24.37
*Aspergillus flavus* Link	24.37					24		24	24	
*A. niger* Tiegh.	24.37	24.37	24	24.37	24	24	24.37			24
*A. ochraceus* K. Wilh.		24							12.24	12.24
*A. terreus* Thom			24				24.37		24	
*A. versicolor* (Vuill.) Tirab.			24.37	24.37					24.37	
*Aureobasidium pullulans* (de Bary) G. Arnaud			24	24.37	24	24.37	24.37		24	12
*Chaetomium globosum* Kunze			24		24					
*Emericella usta* (Bainier) Pitt and A.D. Hocking				24.37	24				24	12.24
*Fusarium sp.* Link						12				
*Humicola lanuginosa Thermomyces lanuginosus* Tsikl.		24.37			24.37		24.37			
*Mucor hiemalis* Wehmer			12.24		12.24		24			
*Penicillium aurantiogriseum* var. aurantiogriseum, Annales de la Société Scientifique de Bruxelles				24						12.24
*P. chrysogenum* Thom				24.37		24.37			12.24	12.24
*P. cyclopium* Westling					24					
*P. decumbens* Thom,					24		24		24	
*Phoma exigua* Desm.		12			12					
*Talaromyces ruber* (Stoll) Yilmaz			24	24		24	24		24	24

**Table 4:** Substrates characteristics.

№	Location of Sampling Point	Substrate Type	Least Moisture Capacity, %	Substrate pH (Aqueous Extract)	CO_3_^2−^ %, Content
1	Beyuk-Dash («Bol’shoj Kamen’») Cave, located in the area of Beyuk-Dash mountain.	Clay sediments.	46	7.6	19
2	Kichik-Dash («Malen’kij Kamen’») Cave, located in the area of Kichik-Dash mountain.	Limestone rock.	4	8.3	43.6
3	An unnamed cave, located near Beyuk-Dash Mountain. The site of an ancient hearth.	Clay sediments from the aphotic zone.	48	7.7	16.7
4	An unnamed cave, located near Beyuk-Dash Mountain.	Clay sediments located beneath tufts of mosses.	36	7.8	14.8
5	An unnamed cave, located near Beyuk-Dash Mountain. The parking lot of an ancient man.	Clay sediments from the aphotic zone.	39	7.8	17.2
6	An unnamed cave (grotto), near Kanizadag mountain.	Limestone rock located beneath tufts of mosses.	3	7.6	43.2
7	An unnamed cave, near Kanizadag mountain. An area near to the ancient rock paintings.	Limestone rock located beneath tufts of mosses.	4	7.8	36.8
8	An unnamed cave, near Kanizadag mountain. An area near to the ancient rock paintings.	Limestone located under biofilms of algae and cyanobacteria.	3	8.4	44.3
9	New small cave located near Kichik-Dash mountain.	Clay sediments from the aphotic zone.	38	7.8	32.6
10	A cave in the area of Yazili Tepe rocks near Kanizadag mountain.	Clay sediments from the aphotic zone.	41	7.6	28.4

**Table 5:** The meaning of Jaccard’s similarity indices (benchmark values of similarity are highlighted).

Algae											
**Microfungi**	**Sample Point**	**1**	**2**	**3**	**4**	**5**	**6**	**7**	**8**	**9**	**10**
	**1**	1	0.38	0.33	0.39	0.2	0.36	0.2	**0.5**	0.14	0.35
	**2**	0.17	1	0.13	0.25	0.31	0.35	0.21	**0.53**	0.21	0.33
	**3**	0.22	0.09	1	0.18	0.09	0.2	0.13	0.33	0.2	0.15
	**4**	0.11	0.1	0.36	1	0.25	**0.44**	**0.44**	0.33	0.18	0.21
	**5**	0.18	0.27	0.38	0.21	1	0.13	0.2	0.26	0.33	0.36
	**6**	0.29	0.11	0.27	**0.44**	0.14	1	**0.41**	0.36	0.29	0.17
	**7**	0.11	0.22	0.5	0.27	**0.42**	0.3	1	0.30	0.2	0.11
	**8**	**0.67**	0	0.11	0	0.09	0.14	0	1	0.26	0.29
	**9**	0.18	0.08	0.38	**0.42**	0.25	0.33	0.31	0.2	1	0.25
	**10**	0.22	0.2	0.33	**0.67**	0.29	**0.4**	0.25	0.11	**0.5**	1

## Data Availability

The datasets generated and analyzed during the current study are available from the corresponding author on reasonable request.

## Supplementary Materials

The following supporting information can be downloaded at: https://www.mdpi.com/article/10.3390/life13010164/s1, Figure S1: Scanning electron micrographs of diatom algae from biofilms; Figure S2: Optical micrographs of algae from biofilms; Figure S3: Proximity matrices; Table S1 Species abundance scores in native communities and when cultured at 12C.
