# Supplementary material for: Biodiversity of Phototrophs and Culturable Fungi in Gobustan Caves
Source: Life (Basel). 2023 Jan 5;13(1):164. doi: 10.3390/life13010164 (PMC9863006; doi:10.3390/life13010164)
Supplement: Supplementary file 1 [file life-13-00164-s001.zip › Figure S1 Scanning electron micrographs of diatom algae from biofilms.docx]

| 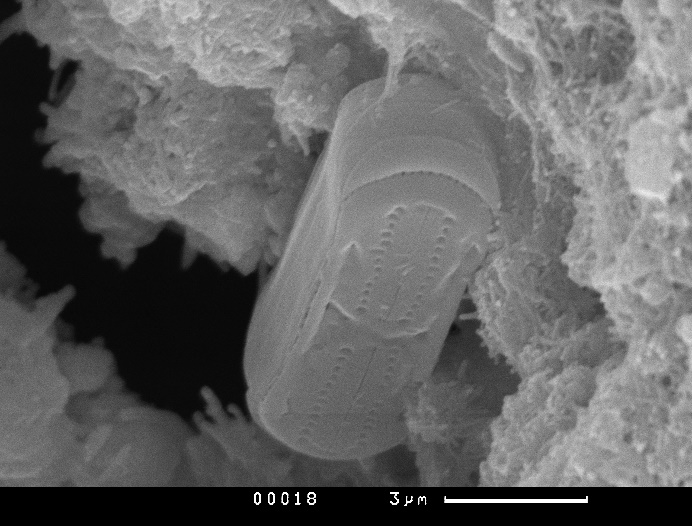 | 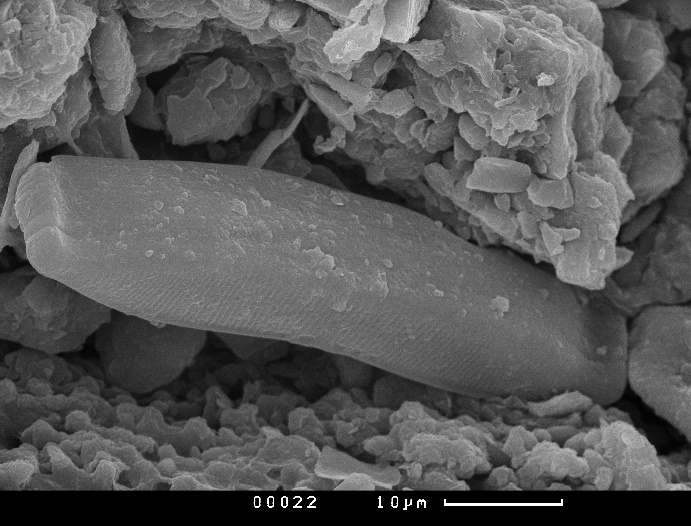 |
| --- | --- |
| (**a**) | (**b**) |

**Figure S1.** Scanning electron micrographs of diatom algae from biofilms: (**a**) *Humidophila contenta*; (**b**) *Hantzschia amphioxys*.
