# Supplementary material for: Biodiversity of Phototrophs and Culturable Fungi in Gobustan Caves
Source: Life (Basel). 2023 Jan 5;13(1):164. doi: 10.3390/life13010164 (PMC9863006; doi:10.3390/life13010164)
Supplement: Supplementary file 1 [file life-13-00164-s001.zip › Figure S2 Optical micrographs of algae from biofilms.docx]

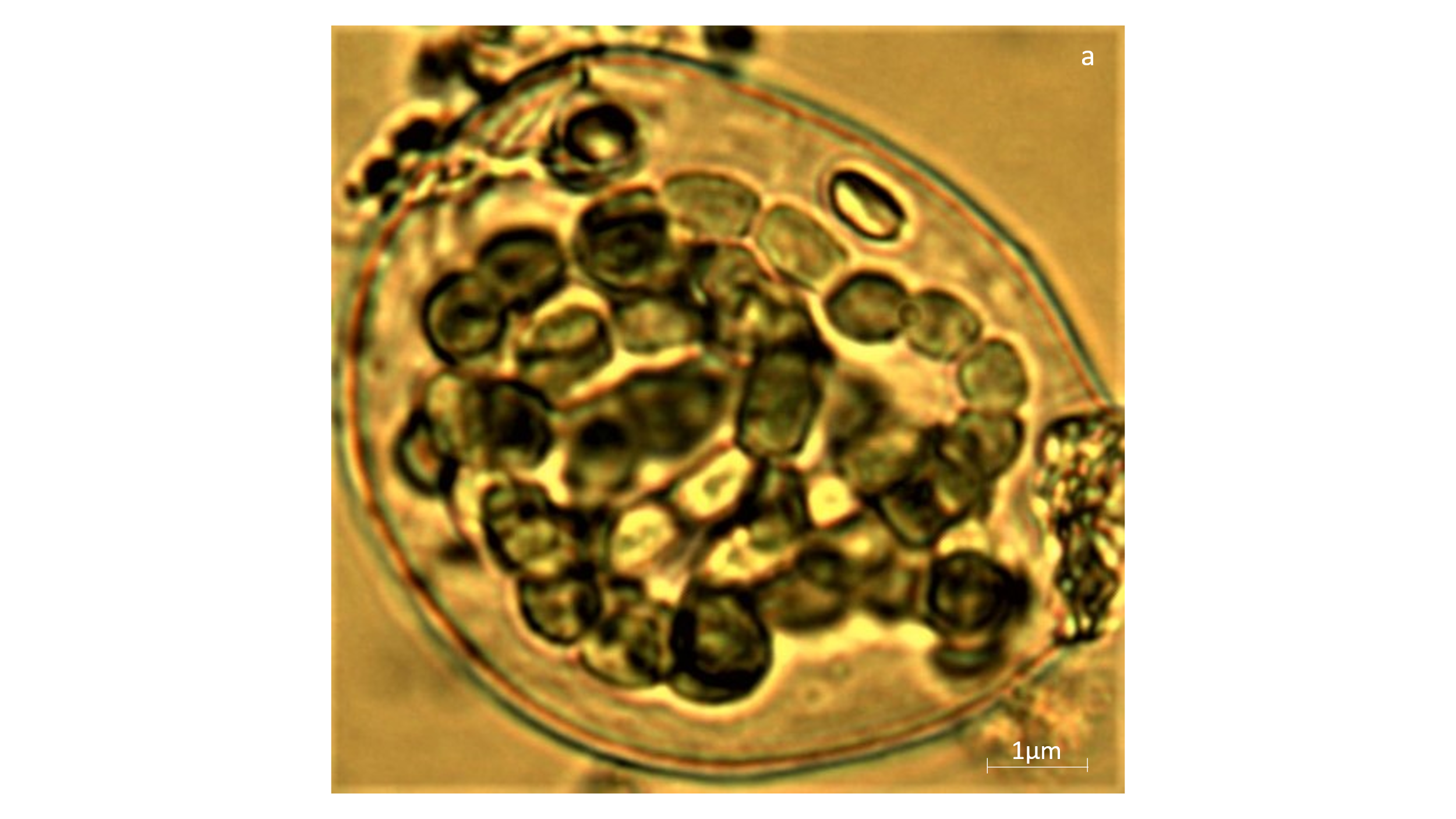


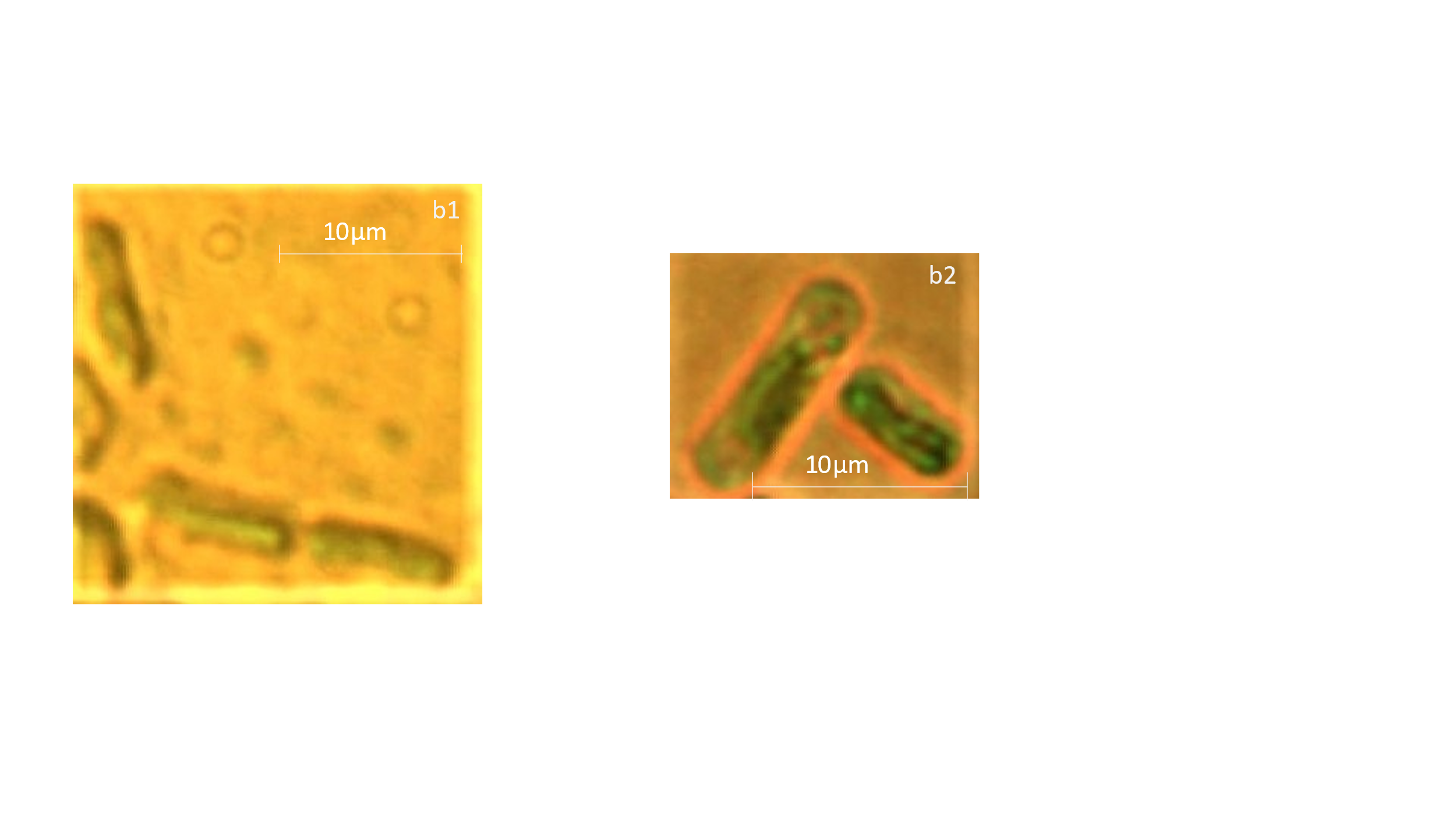


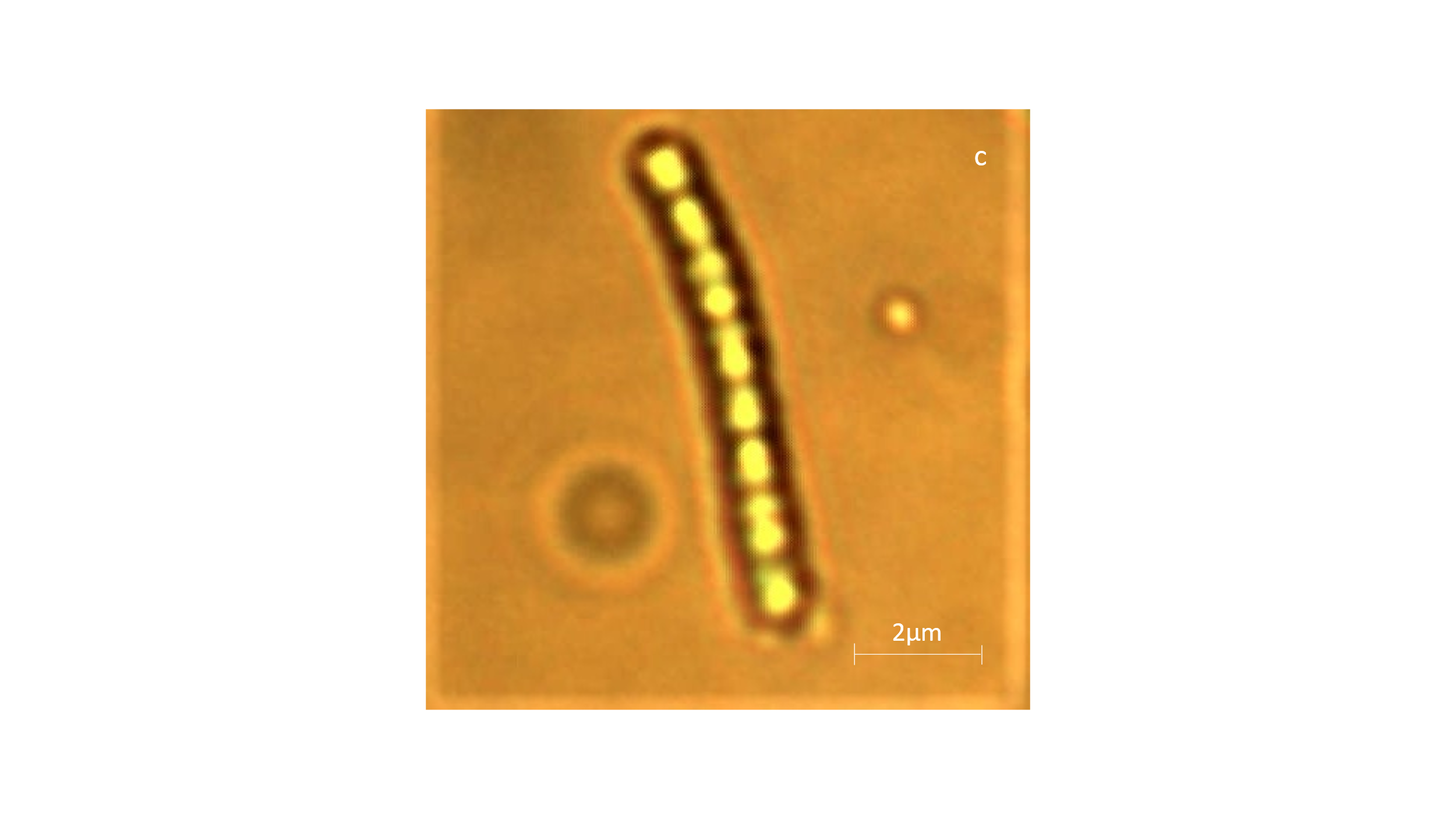


**Figure S2.** Optical micrographs of algae from biofilms: (**a**) *Nostoc microscopicum*; (**b1**,**b2**) *Stichococcus bacillaris*; (**c**) *Planktolyngbya limnetica*.
