# Supplementary material for: Biodiversity of Phototrophs and Culturable Fungi in Gobustan Caves
Source: Life (Basel). 2023 Jan 5;13(1):164. doi: 10.3390/life13010164 (PMC9863006; doi:10.3390/life13010164)
Supplement: Supplementary file 1 [file life-13-00164-s001.zip › Figure S3 Proximity matrices.docx]

a
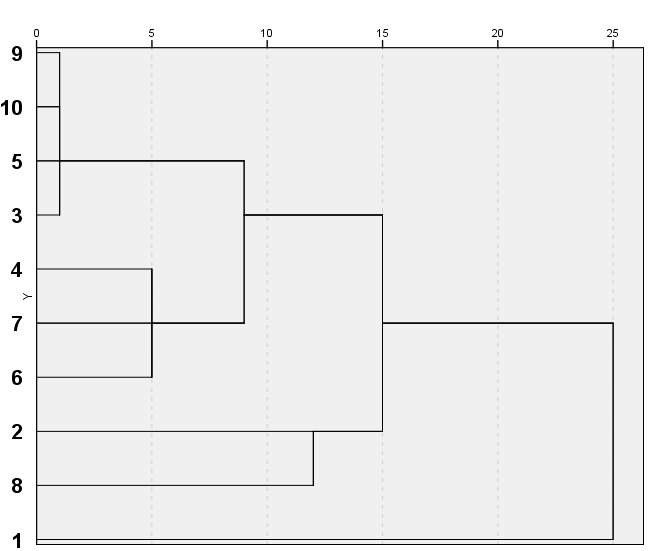
 b
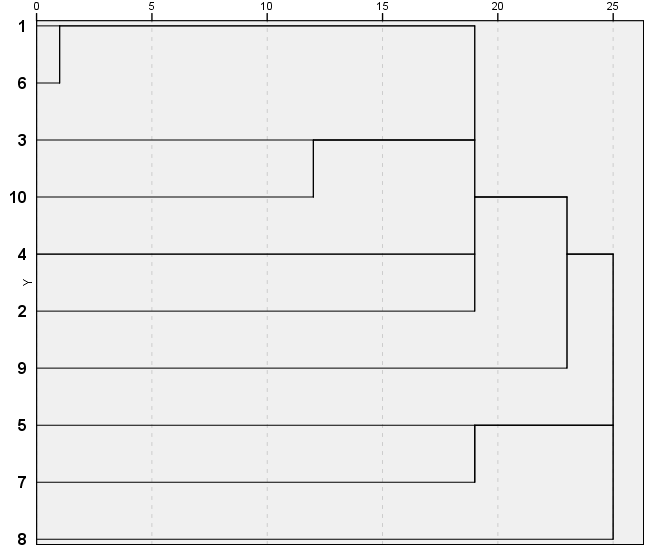


**Figure S3.** Proximity matrices: (**a**) - Matrix of the proximity of the algoflora of the studied samples, estimated using the Euclidean distance; (**b**) - Matrix of proximity of mycobiota of the studied samples, estimated using the Euclidean distance.
